# MiRNA expression profiling and emergence of new prognostic signature for oral squamous cell carcinoma

**DOI:** 10.1038/s41598-021-86316-w

**Published:** 2021-03-31

**Authors:** Christo Rajan, V. G. Deepak Roshan, Imran Khan, V. G. Manasa, Iris Himal, Jayasree Kattoor, Shaji Thomas, Paturu Kondaiah, S. Kannan

**Affiliations:** 1 Division of Cancer Research, Regional Cancer Centre, Thiruvananthapuram, Kerala 695011 India; 2 Department of Molecular Reproduction, Development and Genetics, Indian Institute of Science, Bangalore, Karnataka 560 012 India; 3 Division of Pathology, Regional Cancer Centre, Thiruvananthapuram, Kerala 695011 India; 4 Division of Surgical Oncology, Regional Cancer Centre, Thiruvananthapuram, Kerala India; 5 Department of Zoology, MarThoma College, Thiruvalla, Kerala 689111 India; 6 Division of Genetics and Cytogenetics, Malabar Cancer Centre, Kannur, Kerala 670103 India; 7 Women’s Malignancies Branch, National Cancer Institute, National Institute of Health (NIH), Bethesda, MD 20892 USA

**Keywords:** Biological techniques, Cancer, Genetics, Molecular biology

## Abstract

Oral squamous cell carcinoma (OSCC), the most common type of head and neck cancers, is associated with high recurrence, metastasis, low long-term survival rates and poor treatment outcome. As deregulated miRNA expression plays a crucial role in malignant transformation and cancer progression, the present study is aimed at profiling the miRNA expression pattern in OSCC and developing a new miRNA prognostic signature for oral cancer. MiRNA expression profiling was performed using MiRNA microarray in 30 tumor and 18 normal samples. MiRNA signature obtained was validated with quantitative real time PCR (qRT-PCR) in 144 tumor and 36 normal samples. The potential targets, clinical implications and prognostic value of the miRNA signature were elucidated by various bioinformatics and statistical analyses. Microarray profiling identified a set of 105 miRNAs to be differentially expressed in OSCC, out of which a subset of 19 most dysregulated miRNAs were validated by qRT-PCR. In silico analysis revealed the signature miRNAs to be involved in various cancer associated pathways. Up-regulation of miR-196a, miR-21, miR-1237 and downregulation of miR-204, miR-144 was associated with poor prognosis of OSCC patients. The mir-196a/miR-204 expression ratio emerged as best predictor for disease recurrence and patient survival. Altogether, our study identified a miRNA signature for OSCC with prognostic significance.

## Introduction

Globally, one in every six deaths is caused by cancer, and in 20 years, its incidence and mortality is expected to grow to 27.5 and 16.3 million cases respectively^1^. With more than half a million new cases reported annually, Head and Neck Squamous Cell Carcinoma (HNSCC) stands as the sixth leading cancer by incidence and eighth by death worldwide^2,3^. The 5-year survival rate of patients with HNSCC is 40–50%^3^. Oral Squamous cell Carcinoma (OSCC) accounts for nearly 95% of all the head and neck cancers with an annual incidence of over 300,000 cases^4^, 62% of which arise in developing countries^5^. In the U.S. population, oral cavity cancer represents only about 3% of malignancies, but in India, it accounts for over 30% of all cancers and hence ranks among the top three types of cancers in India^6^. It is also the leading cause of cancer death among men in India^2^. Low socio-economic status, illiteracy, lack of awareness, limited access to specialized treatment and diagnosis results in more than 48% of these cases being diagnosed in later stages, i.e. III and IV^7^. Despite all our advancements, treatment failures remain frequent, calling for better management of disease and prognostic tools.

As a family of super-regulatory small non-coding RNAs, microRNAs (miRNA) have regulatory roles in diseases via regulating expression of protein-coding target genes at both post-transcriptional and translational levels. MiRNAs work by targeting complementary gene transcripts either sequestering them in the cytoplasm or degrading them out-right depending on the degree of complementarity of the target transcript to the miRNA seed region^8^. They have high specificity in expression with regards to tissue type and disease characteristics^9^. It has been demonstrated that miRNA expression profiles have better accuracy in disease classification than mRNA expression profiles^10,11^. A myriad of miRNAs has proven to be differentially expressed across the many studies addressing OSCC. Despite the growth in literature on miRNAs and their specific expression status across tissue and disease types, each new study brings up a new profile with very few common signatures. Ethnic variations in the study subjects have been found to be a major contributor to this difference^11^. Most of the present literature on oral cancer specific miRNAs come from European and American study subjects comprising mostly of Caucasians, Hispanics and Blacks, whereas the major burden of the disease is on developing countries^5^ especially the Indian Peninsula. Besides a few tissue level miRNA expression studies, majority of the data is derived from oral cancer cell lines^12^, inadequate to provide a significant picture on miRNA expression status. Addressing these issues, our study is conducted in a cohort of 144 OSCC tissue samples obtained from a region with one of the highest prevalence of OSCC in the whole of Asia. With this study we hope to bring forth a miRNA expression signature which could prove to be an effective diagnostic and prognostic marker and a potential therapeutic target.

## Results

### miRNA microarray and the expression pattern in OSCC

**Figure 1:**
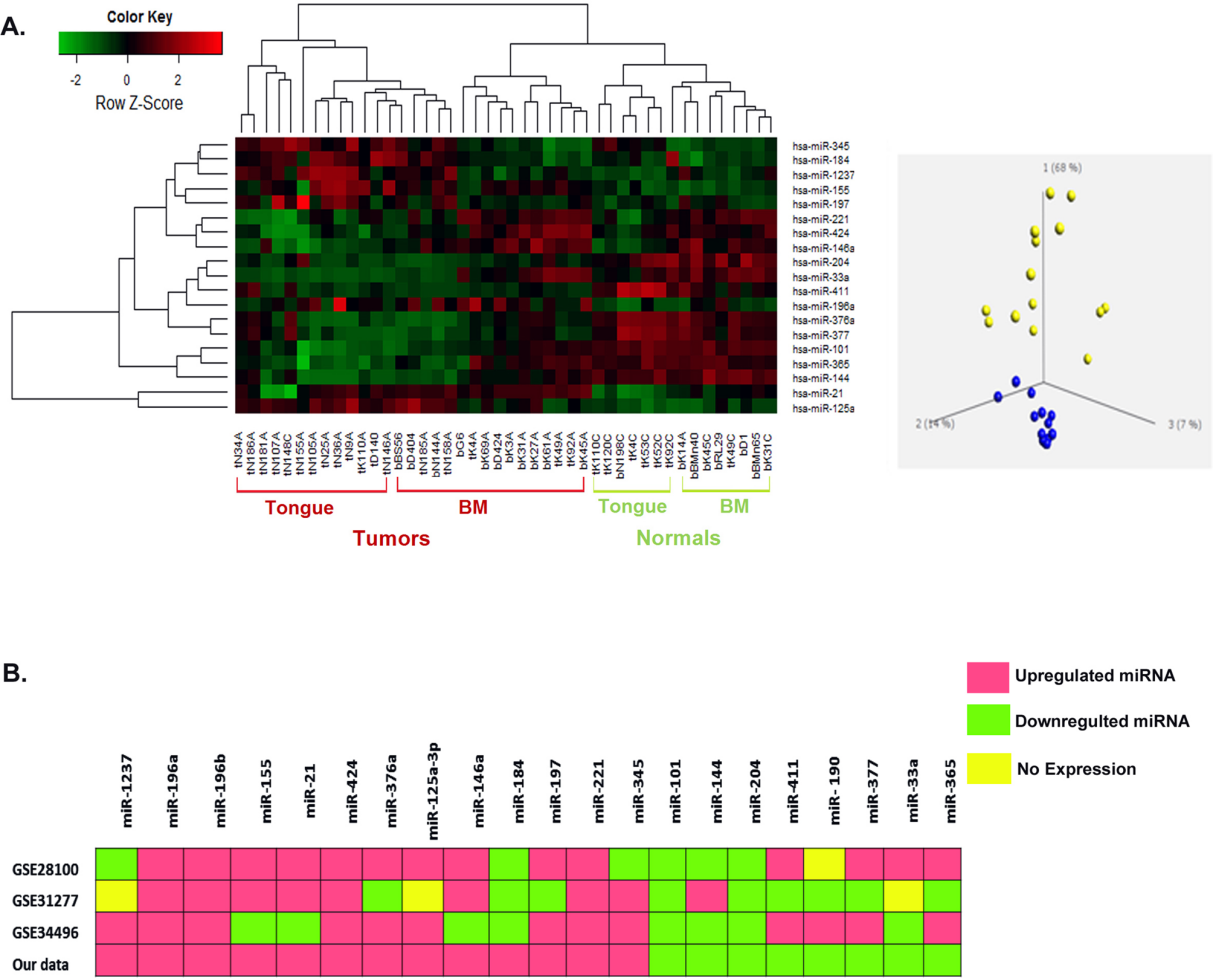
(**A**) Supervised hierarchical clustering using signature miRNAs differentiating samples site-wise into distinct Tongue and Buccal mucosa clusters with cut off Fold Change > 1.5 and *p* value < 0.01. In the heatmap, samples are labelled with prefix ‘t’ for Tongue and ‘b’ for Buccal Mucosa (BM) and the suffix ‘C’ denotes Normals while the rest represents Tumor samples. (**B**) Heatmap showing the expression status of signature miRNAs in three external datasets compared to our profile; GSE28100 (USA, tongue), GSE31277 (Brazil, OSCC) and GSE4496 (USA, OSCC). Pink boxes represent upregulated miRNA, green boxes represent downregulated miRNA and yellow boxes indicates absence of miRNA expression.

Our primary focus was to identify differentially expressed miRNAs (DE miRNAs) in oral carcinoma with respect to normal mucosa, in a set of 48 samples which included 30 tumors and 18 normal controls. Differential expression analysis showed around 105 miRNAs to be significantly dysregulated in oral carcinoma with a *p* value < 0.05 and fold change > 1.5. Narrowing down for the 50 most significant DE miRNAs, unsupervised hierarchical clustering and PCA broadly clustered tumor from normal. Filtering further for significance (cut-off of *p* value < 0.01), a 19 miRNA list split the heatmap into tumor and a normal cluster clearly grouping them apart as in the corresponding PCA (Fig. 1A, Table 1). Intraoral tumor site-wise grouping also noticed in this clustering, precise separation of clusters into Tongue and Buccal Mucosa tumors and normals, highlighting disease as well as site specific expression of microRNAs (Fig. 1A, Supplementary Figure 1). In addition, pathway analyses of miRNAs downregulated and upregulated in Tongue and Buccal Mucosa tumors indicated these significantly deregulated miRNAs to be targeting distinct cancer associated pathways (Supplementary Table 2). Thus, this 19 DE miRNA subset may house possible candidates with clinical implications in oral carcinoma.

**Table 1:** Differentially expressed miRNAs in oral carcinoma derived by *p* value < 0.01 filtering.

miR.id	miRbase	Chromosome	Tumor vs Normal	Tongue	BM	fdr *p* value
hsa-miR-21-5p	MIMAT0000076	17q23.1	2.081	2.368	1.852	0.000
hsa-miR-125a-3p	MIMAT0004602	19q13.41	1.641	1.923	1.486	0.000
hsa-miR-196a-5p	MIMAT0000226	17q21.32	1.228	1.089	1.728	0.004
hsa-miR-155-5p	MIMAT0000646	21q21.3	1.026	1.22	1.201	0.002
hsa-miR-1237-3p	MIMAT0005592	11	0.68	1.148	0.174	0.007
hsa-miR-302c-3p*	MIMAT0000717	4q25		0.67		0.004
hsa-miR-184	MIMAT0000454	15q25.1	0.606	0.699	0.737	0.004
hsa-miR-197-3p	MIMAT0000227	1p13.3	0.547	0.767	− 0.03	0.002
hsa-miR-146a-5p	MIMAT0000449	5q34	0.309	0.348	0.826	0.001
hsa-miR-221-3p	MIMAT0000278	Xp11.3	0.22	0.294		0.010
hsa-miR-424-5p	MIMAT0001341	Xq26.3	0.086	-0.124	0.688	0.004
hsa-miR-345-5p	MIMAT0000772	14q32.2	0.031	0.242	− 0.404	0.006
hsa-miR-411-5p	MIMAT0001080	7p15.2	− 0.187			0.000
hsa-miR-365a-3p	MIMAT0000710	16p13.12	− 1.22	− 1.59	− 0.576	0.006
hsa-miR-33a-5p	MIMAT0000091	22q13.2	− 1.957		− 0.704	0.002
hsa-miR-101-3p	MIMAT0000099	1p31.3	− 2.384	− 2.742	− 1.788	0.000
hsa-miR-204-5p	MIMAT0000265	9q21.12	− 2.572		− 2.691	0.000
hsa-miR-377-3p	MIMAT0000730	14q32.31	− 2.872	− 3.994	− 2.099	0.003
hsa-miR-376a-3p	MIMAT0000729	14q32.31	− 3.265	− 4.095	− 2.66	0.001
hsa-miR-144-3p	MIMAT0000436	17q11.2	− 4.51	− 5.447	− 3.426	0.001
hsa-miR-196b-5p*	MIMAT0001080	7p15.2		1.323		0.000
hsa-miR-190-5p*	MIMAT0000458	15q22.2	− 1.31	− 1.531		0.000

Log_2_ fold change values shown.

*miRNAs not part of the 19 signature, expressed in Tongue SCC alone.

### Real time PCR testifies the miRNA microarray signature profile

TaqMan quantitative Real-time PCR was used to verify and validate the 19 DE miRNA signature. The Ct values obtained were normalized using Mean Expression Value normalization method^13^. We validated the 19 DE miRNAs using relative quantification in a larger cohort of 180 (144 OSCC and 36 normal) tissue samples, which we split into a tester (n = 25 paired tumor and normal) and trainer (n = 120 tumors and 36 normals) groups. We found that the fold expression of miRNAs obtained through qRT-PCR, corroborated all the 19 miRNAs assayed (Table 2) i.e. the tester vs array (Pearson correlation = 0.711, *p* = 0.001), trainer vs microarray (Pearson correlation = 0.726, *p* = 0.001) and tester versus trainer (Pearson correlation = 0.936, *p* = 0.001) strongly validating our miRNA microarray expression profile.

**Table 2:** Validation table.

Name	miRbase access	Array	Tester25	Trainer120
hsa-miR-21-5p	MIMAT0000076	2.081	2.627	1.303
hsa-miR-125a-3p	MIMAT0004602	1.641	1.091	1.847
hsa-miR-196a-5p	MIMAT0000226	1.228	5.099	4.592
hsa-miR-155-5p	MIMAT0000646	1.026	1.185	2.615
hsa-miR-1237-3p	MIMAT0005592	0.68	1.991	2.544
hsa-miR-184	MIMAT0000454	0.606	0.463	0.524
hsa-miR-197-3p	MIMAT0000227	0.547	− 0.179	0.279
hsa-miR-146a-5p	MIMAT0000449	0.309	1.373	1.041
hsa-miR-221-3p	MIMAT0000278	0.22	0.397	0.638
hsa-miR-424-5p	MIMAT0001341	0.086	1.283	1.353
hsa-miR-345-5p	MIMAT0000772	0.031	− 0.583	0.207
hsa-miR-411-5p	MIMAT0003329	− 0.187	− 0.803	− 1.478
hsa-miR-365a-3p	MIMAT0000710	− 1.22	− 0.342	− 0.576
hsa-miR-190a-5p	MIMAT0000458	− 1.31	− 1.902	− 2.674
hsa-miR-33a-5p	MIMAT0000091	− 1.957	− 0.456	− 1.095
hsa-miR-101-3p	MIMAT0000099	− 2.384	− 1.321	− 1.884
hsa-miR-204-5p	MIMAT0000265	− 2.572	− 4.194	− 4.097
hsa-miR-377-3p	MIMAT0000730	− 2.872	− 3.259	− 3.709
hsa-miR-376a-3p	MIMAT0000729	− 3.265	− 1.571	− 2.094
hsa-miR-144-3p	MIMAT0000436	− 4.51	− 2.587	− 2.527
hsa-miR-196b-5p	MIMAT0001080		3.141	3.991

Showing Log_2_ Fold change values of significant mRNAs across both platforms—Array (miRNA microarray) and Real-Time PCR (tester 25 and trainer 120).

### In-Silico analysis of validated miRNA signature

The signature miRNA list was dichotomized into up-regulated (upmiRs) and down-regulated (downmiRs) and subjected to bio-informatic target prediction using DIANA microT. This was followed by enrichment of target genes sets in DAVID to look at the putative target genes for each of the two groups. Pathway prediction for both up (supplementary Table 2a) and down regulated (supplementary Table 2b) miRNA lists was done using DIANA miRPath and KEGG (Kyoto Encyclopaedia of Genes and Genomes). TGFb-SMAD pathway (*p* = 0.001), endocytosis (*p* = 0.001), ErbB signalling pathway (*p* = 0.01) and ECM-receptor interaction pathway (*p* = 0.01) was regulated by the upmiRs and MAPK signalling (*p* = 0.001), PI3K-Akt signalling pathway (*p* = 0.001), pathways in Cancer (*p* = 0.001) and Wnt signalling pathways (*p* = 0.004) involved the downmiRs. Pooling the common miRNAs in these pathways we arrived at a 13 miRNA subset containing up-regulated miR-1237, miR-155, miR-196a, miR-196b, miR-21 and miR-424 and down-regulated miR-411, miR-144, miR-190, miR-101, miR-204, miR-376a and miR-377, taken up for further investigation. Among these, over-expression of miR-1237 and down-regulation of miR-411 and miR-377 was fairly consistent without much supportive literature, especially in OSCC.

### Meta-analysis depicts the global status of our DE miRNAs in oral cancer

To check whether the DE miRNA was unique to our cohort, we looked at their expression in three oral cancer miRNA microarray datasets. All the datasets were extracted from Gene Expression Omnibus (GEO) by querying for keyword “Oral Cancer miRNA” where, out of the 22 results, 3 interested us; GSE 28100:^14^ GSE31277:^15^ and GSE 34496:^16^ (Supplementary Table 3). Together these datasets contained 83 tumors and 31 normal cases. Differential expression analysis showed that the 13 signature miRNAs were significantly dysregulated in these datasets as well. MiR-196a, miR-196b and miR-424 were consistently upregulated and miR-101 and miR-204 were shown to be consistently down-regulated in all the three datasets, while the rest of the miRNAs showed expressional variation (Fig. 1B).

### The clinical implications of the signature miRNAs

**Figure 2:**
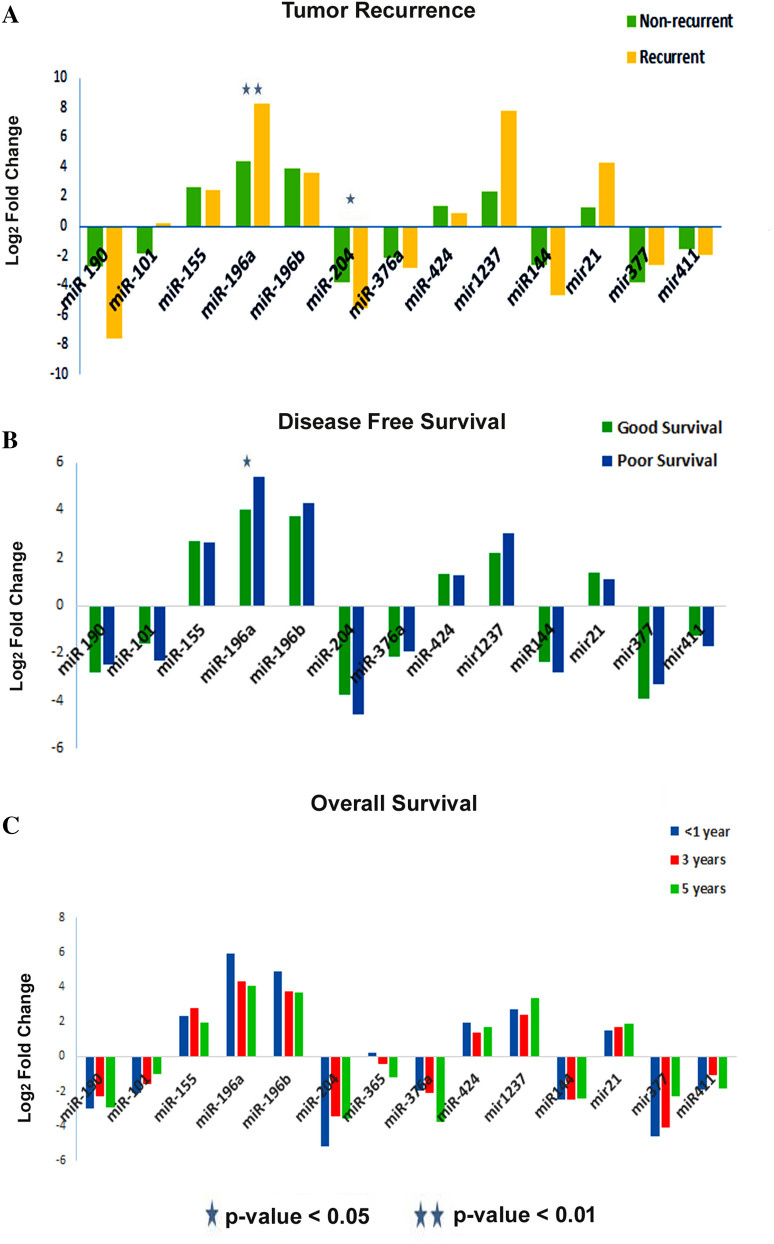
Validated expression profiles of signature miRNAs compared to Clinico-Pathological characteristics of patient cohort. (**A**) Tumor recurrence (Green and orange bars represent non-recurrent and reccurent tumors respectively) (**B**) Disease Free Survival (green bars for good and blue bars for poor survival) and (**C**) Overall survival (Blue bars for average < 1 year, red for average 3 years and green for average 5 years).

Comparing recurrent versus non-recurrent tumors, we noticed a sharp upregulation of miR-196a (*p* = 0.009), miR-1237 (*p* = 0.08) and miR-21 (*p* = 0.04) in recurrent samples and a down regulation of miR-190 (*p* = 0.07), miR-204 (*p* = 0.03) and miR-144 (*p* = 0.05) (Table 2, Fig. 2A). Surprisingly we found that up-regulation of miR-196a (*p* = 0.02), and the downregulation of miR-204 (*p* = 0.06) indeed associated with poor DFS (Table 3, Fig. 2B). To correlate miRNA expression with over-all survival, we split the parent cohort into low overall survivors (≤ 1 year), intermediate (1–3 year) and good survivors (≥ 5 years). We found that miR-196b (*p* = 0.014) was highly up-regulated in the low survival group, gradually decreasing expression with increasing survival, as was the down-regulation of miR-376a (*p* = 0.08), gradually making up with increasing overall survival (Table 3; Fig. 2C). Peri-neural Invasion (PNI) and tumor depth are two prognostic indicators of cancer progression. Up-regulated miR-196a, miR-196b and miR-424 and under-expressed miR-190, miR-204 and miR-377 associated with PNI positivity (Table 3, Supplementary Figure 2a). Interestingly, Mann–Whitney U test associated the over-expressions of miR-1237 (*p* = 0.05) which showed a sharp increase in low depth, early tumors, miR-21 (*p* = 0.09) and down-regulation of miR-204 (*p* = 0.003), miR-411 (*p* = 0.06) to tumor depth (Tables 3, 4, Supplementary Figure 2b). Thus, miRNAs 196a, 21, 1237, 204 and 144 were shown to have some prognostic significance for oral carcinomas. Kaplan–Meier survival curves were plotted for these five miRNAs to check for association between their altered expression levels and overall survival (OS) as well as disease free survival (DFS). Interestingly, miR-196a (log rank *p* = 0.027 (OS) *p* = 0.023 (DFS) and miR-204 (log rank *p* = 0.06(OS) *p* = 0.08) (DFS)) showed the most significant association with patient survival (Table 4; Supplementary Figure 3). We also computed the disease predictive power of each of the 5 miRNAs using ROC curve. Of the 5 miRNAs tested miR-196a (AUC = 0.848, specificity = 83.78 and sensitivity = 77.08) and miR-204 (AUC = 0.842, specificity = 94.95 and sensitivity = 79.44) gave the best predictive potential (Supplementary Table 4).

**Table 3:** 13 Signature miRNA expression and association with clinico-pathological factors using non-parametric Mann-Whitney U test.

Variable	miR-190	miR-101	miR-155	miR-196a	miR-196b	miR-204	miR-376a	miR-424	miR-1237	miR-144	miR-21	miR-377	miR-411
**Tumor depth (n = 61)**													
U	328	294	320	373	369	**191**	278	340	**252**	334	**271**	364	**258**
*p* value	0.459	0.198	0.385	0.987	0.936	**0.003**	0.123	0.584	**0.05**	0.52	**0.09**	0.0872	**0.062**
**Perineural invasion (n = 120)**													
U	341	363	**312**	409	329	373	370	347	367	417	406	432	429
*p* value	0.19	0.32	**0.084**	0.741	0.138	0.396	0.372	0.221	0.349	0.829	0.708	1	0.966
**Tumor recurrence (n = 120)**													
U	**929**	1017	1096	**718**	927	**798**	1066	1066	**845**	**812**	**799**	951	1069
*p* value	**0.072**	0.558	0.957	**0.009**	0.234	**0.039**	0.798	0.798	**0.081**	**0.052**	**0.04**	0.303	0.874
**Disease free status (n = 118)**													
U	1442	1528	1596	**1237**	1393	1342	1592	1589	1480	1614	1488	1582	1657
*p* value	0.192	0.405	0.645	**0.026**	0.116	0.064	0.63	0.618	0.273	0.718	0.293	0..591	0.9
**Overall survival (n = 119)**													
U	1569	1446	1631	1481	**1231**	1391	**1365**	1578	1578	1632	**1322**	1677	1543
*p* value	0.548	0.204	0.793	0.154	**0.014**	0.116	**0.087**	0.582	0.582	0.798	**0.051**	0.991	0.458

*PNI *perineural invasion.

Bold values denotes the factors statistically siginificant with *p* value < 0.10.

**Table 4:** Log rank survival statistic of the five recurrence specific miRNAs.

miRNA	miR-196a	miR-21	miR-1237	miR-204	miR-144
**Overall survival**					
χ^2^	4.876	1.138	0.371	2.909	1.28
Log rank *p* value	0.027	0.286	0.542	0.081	0.258
**Disease free survival**					
χ^2^	5.159	0.964	0.482	3.351	1.119
Log rank p value	0.023	0.326	0.488	0.067	0.29

*OS* overall survival, *DFS* disease free survival.

miRNA expression ratios have demonstrated a better potential and an increase in predictive power^17,18^. We took the expression ratio by dividing the relative expression values of the up-regulated miR-196a with the down-regulated miR-204. i.e. miR-196a/miR-204. A Kaplan Meier curve using the miRNA expression ratio proved miR-196a/miR-204 ratio a better predictor of patient survival where samples having miR-196a/miR-204 ratio < 1 survived better than samples with ratio > 1 [Log Rank *p* = 0.066 (OS) and *p* = 0.063 (DFS)] (Supplementary Figure 4a) i.e. survival decreased with higher miR-196a/miR-204 ratio. ROC curve with AUC = 0.888, Specificity = 91 and Sensitivity = 83, demonstrated the excellent predictory power of miR-196a/miR-204 ratio, to demarcate between aggressive and non-aggressive oral tumors (Supplementary Figure 4b).

### The translational potential of miR-196a/miR-204 ratio as a prognostic marker

**Figure 3:**
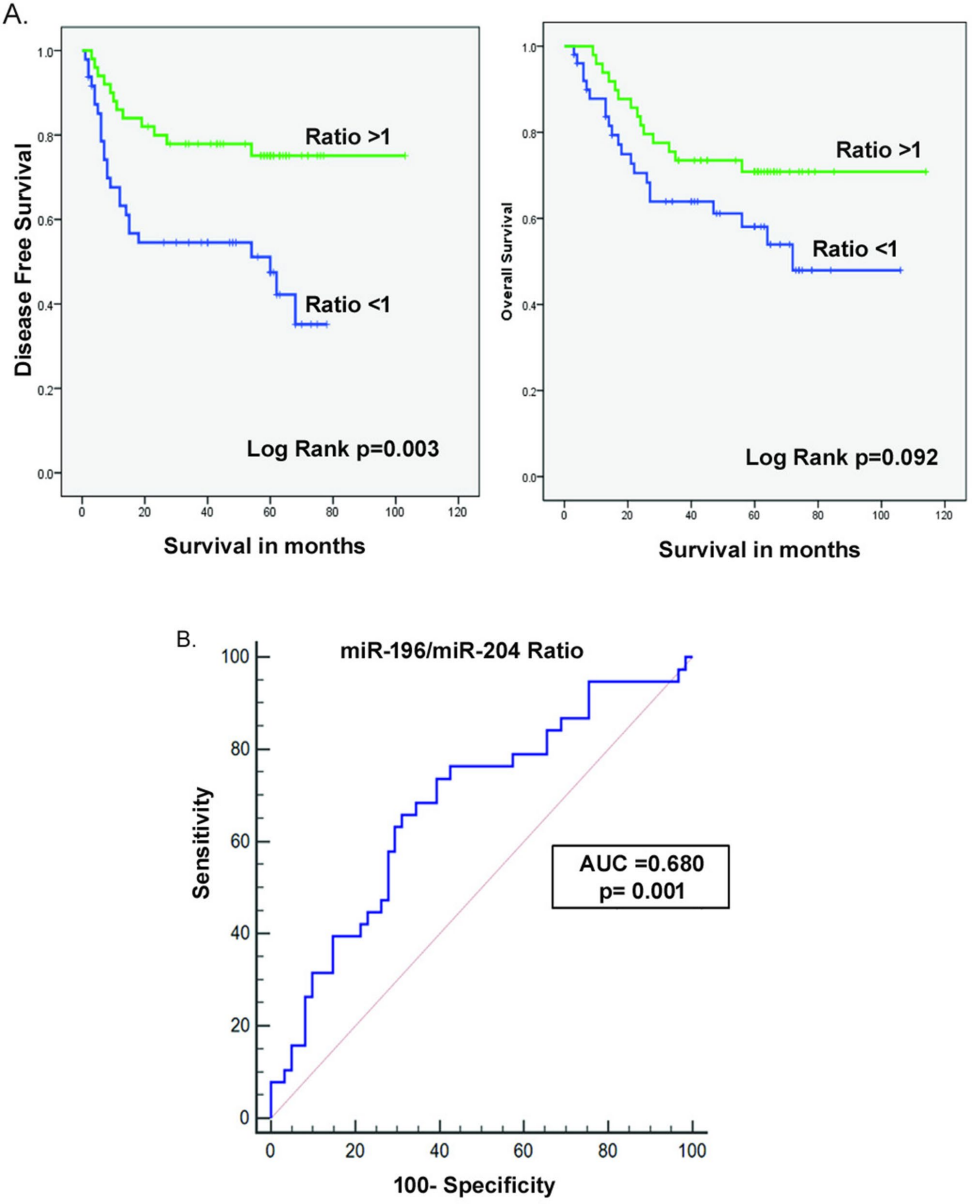
(**A**) Expression ratio (normalized Ct) of miR-196a/miR-204 and its association with Disease-free and Overall survival. (**B**) ROC demonstrates the predictory power of miR-196a/miR-204 expression ratio.

For all above analysis, normalization was done using global mean normalization method^13,19^ as it seems to be superior over internal control U6 normalization. However, it is not practical in measuring one or two microRNAs for routine diagnostic or prognostic purposes. In such cases, internal control normalization is more suitable and practical. Similarly the calculation of fold change is also not possible as it is difficult to have corresponding normal tissue in all cases. The absolute copy number of transcripts is ideal in such situations, but known standards of miRNA of interest is required for absolute quantitation. Hence, more practical and feasible way is to calculate the normalized Ct of miRNAs of interest using an internal control like U6 or any other ubiquitous miRNA. Hence, we also validated our miR-196a/miR-204 ratio in available tumour total RNA samples with U6 normalization. In total, we calculated normalized dCt for miR-196a and miR-204 in 100 oral cancer samples using U6 as internal control. Then the expression ratio was calculated by dividing the normalized dCt value of the up-regulated miR-196a with the down-regulated miR-204. i.e. miR-196a/miR-204. Then the median of the miR-196a/miR-204 ratio was calculated to dichotomize the data to high and low. The median for the miR-196a/miR-204 ratio was 0.74 and for the convenience it was taken as 0.75. The ratio less than 0.75 was considered as low and the ratio equal to or above 0.75 was taken as high ratio. Then the prognostic significance of the miR-196a/miR-204 ratio was evaluated using Kaplan Meier curve. The results showed that the miRNA expression ratio has significant association with the DFS of oral cancer patients. The high miR-196a/miR204 ratio demonstrated better disease free survival than patients with low ratio (Log Rank *p* = 0.003) (Fig. 3A). However, its relation with the overall survival of patients is marginally significant (Log Rank *p* = 0.092). ROC curve analysis also demonstrated the excellent predictory power of miR-196a/miR-204 ratio, with AUC = 0.680, *p* = 0.001, specificity = 69 and sensitivity = 66, to demarcate patients with good and poor disease free survival (Fig. 3B). Along with miR-196a/miR-204 ratio, significance of other classical clinical prognostic factors with DFS and OS were also evaluated by both univariate (Kaplan Meier/Log Rank) and multivariate (Cox’s propotional hazard) methods (Supplementary Table 5). With respect to DFS, miR-196a/miR-204 ratio alone was significant in both univariate and multivariate analysis. However, with respect to OS, the T-status and composite stage were shown to be significant in univariate analysis, but not in multivariate model. The miR-196a/miR-204 ratio was marginally significant with OS both in univariate and multivariate analysis. Thus the independent prognostic influence of the miR-196a/miR-204 ratio on survival of oral cancer patients was shown over other clinical factors.

### Potential targets for the signature miRNAs

MiRNAs function by regulating the expression of their target genes and therefore the potential targets of the differentially expressed miRNAs were explored. All the miRNAs had experimentally validated miRNA target list in mirtarbase. The miRNA targets with the maximum number of validation methods and reported publications were selected for correlation analysis. The top three genes which showed a strong negative correlation with the miRNA expression were identified (Supplementary Table 6). The top three targets for miR-196a-5p are SPRR2C, ANXA1, S100A9 and miR-204-5p are CDC42, RAB22A, EZR. Furthermore, FAM69C, PACS2, SPOP were identified to have a negatively correlation with miR-1237-3p expression. In addition, DIANA MirPath analysis revealed Hippo signalling pathway to be the major pathway regulated by miR-196a/miR-204 (Supplementary Figure 5).

## Discussion

The expression pattern of the 19 microarray DE miRNAs packed enough discriminatory power to distinctly cluster the samples into site specific normals and tumors as evidenced by the hierarchical cluster. Further they separated the tongue and buccal mucosa samples into respective normals and tumors, indicating that these signatures were equally demarcative in both the oral sites. Similarly, clinico-pathological attributes like tumor stage and recurrence were also well represented by up-regulated miR-1237 and down-regulated miR-377 and miR-144. The tumor specific over-expression of miR-1237 is a novel report while other signature miRNAs like miR-21, miR-424, miR-196a, miR-196b and miR-204 have been studied and reported in human cancers including OSCC^20–23^.

The expression profiles obtained through miRNA microarray needs to be validated by an independent profiling method and TaqMan qRT-PCR with its detection sensitivity, sequence specificity and reproducible quantitation stands ahead^19^. But the accuracy of TaqMan chemistry could be seriously misinterpreted by an incorrect and unsuitable normalizing gene^24^. Mean expression value normalization was shown to outperform standard small RNA controls in stability and bring out the true biological changes in both tissues and cell lines^13^ and hence employed. The miRNA expression obtained through qRT-PCR, corroborated all the 19 miRNAs assayed i.e. the tester vs array providing a strong validation of our miRNA expression profile. Adding a touch of novelty to the study, the over-expression of miR-1237 was highly indicative of a potential tumor signature and one among the most consistently dysregulated miRNA in our analysis. The up-regulation was much more pronounced in tongue with serious role in tumor depth. In-silico target prediction for miR-1237 revealed putative target sites on multiple genes involved in various cancer associated pathways like; transcriptional mis-regulation in cancer, homologous recombination, endocytosis and PI3K-AKT signalling. Hence there is strong enough reason to consider miR-1237 as an onco-miR especially in the context of oral cancer. miR-1237 has previously been shown to be epigenetically silenced in colo-rectal cancer and HCT-116 cell lines^25^ but upregulated in acute lymphoblastic leukaemia cells cultured in human derived osteoblast cell niche^26^.

Meta-analysis using three independent miRNA microarray datasets GSE8100, GSE3177 and GSE34496 helped re-affirm the expression profile of the DE miRNAs in this study. Together, the three datasets include miRNA expression data from 128 OSCC specimen not biased by any conditions or treatments. The heatmap shows the similarity of expression of miRNAs across the datasets compared to our samples. GSE31277 shows the most expression similarity to our set of samples presumably due to the Asian-Hispanic ethnicity of the Brazilian cohort as against the other two American groups. MiR-196a, miR-196b and miR-424 showed steady and consistent over-expression and miR-101 and miR-204 showed down-regulation in all three cases. Hence these miRNAs warrant special attention due to their onco-specific expressional stability even across different platforms and study conditions.

MiRNA expression profiles have far reaching implications as biomarkers in disease models especially in cancer. miR-204-5p is reported to inhibit proliferation of gastric and colorectal cancer cells by down regulation of RAB22A^27,28^. miR-204-5p acts through cdc42 to aid the invasion of EBV-associated nasopharyngeal carcinoma cells^29^. miR-196a-5p has shown to inhibit ANXA1 and enhance breast cancer cell growth^30^. miR-196a, miR-21, miR-1237, miR-204 and mR-144 were found to be associated with poor disease prognosis and poor DFS in our samples. Aberrant expression of miR-21 has already been suggested as putative diagnostic biomarker for OSCC^31^ and an independent predictor of poor survival for patients with tongue SCC^32^. MiR-144 has also been assigned with tumor suppressive function in lung cancer, and has been reported to inhibit proliferation, induce apoptosis and autophagy upon supplementation in lung cancer cells^33^ and down regulated in oral cancer^22^. Both miR-196a and miR-196b have been proposed as biomarkers in oral cancer management and their overexpression was found to promote oral cancer cell migration, invasion and lymph node metastasis^34^. Similarly, miR-204 is a well reported dis-regulated miRNA with tumor-suppressive functions and is consistently found down–regulated in oral cancer and regulates cancer stemness^20,35^. In fact, Kaplan–Meier survival analysis revealed the association of miR-196a and miR-204 individually with overall (OS) and disease-free survival (DFS) in our sample set. Additionally, ROC curves plotted to look into the predictory potential of the 5 miRNAs yielded results very much in favour of miR-196a and miR-204 dysregulations.

Previously many groups have tried the combinatory potential of miRNA expression ratios in disease prediction^17,18,36,37^. Avissar et al. showed how expression ratio of miR-221: miR-375 distinguished oral tumors from normal and their corresponding diagnostic and prognostic potential^17^. Neely et al. showed how miR-21:miR-205 ratio characterized invasive bladder cell lines from normal non-invasive cell lines by a tenfold margin^38^. It has also been reported in veterinary medicine where miR-17-5p/miR-155 was proposed as a new grading tool for canine splenic lymphomas as it correlated with WHO grading^36^. Similarly, in our study we combined the individual strengths of miR-196a and miR-204 by plotting their expression ratio. The disease predictory power of miR-196a/miR-204 ratio was strengthened by its strong association with survival as manifested by the Kaplan–Meier curve. To be noted here, is the fact that computing the miRNA expression ratio, slightly enhanced the AUC than the individual profiles of miR-196a and miR-204 positively boosting predictive outcome. Tsai et al.^38^ also showed that combined expression signatures of miR-375, miR-204 and miR-196a are promising biomarkers for the diagnosis, prognosis and treatment of OSCC. However, they have not shown its prognostic significance as in the present study with follow-up data. The miRNA expression ratios for the other three miRNAs; miR-1237, miR-21 and miR-144 were not calculated as their association with patient survival was relatively inferior, hence questioning the credibility of their expression ratios if at all. Since miR-196a and miR-204 were both dysregulation markers for poor prognosis, the predictory power of their expression ratio has a potent prognostic effect, where patients expressing a miR-196a/miR-204 ratio < 1 can be predicted to proceed to increased morbidity. Examining their profiles in pre-neoplastic lesions, early onset tumor samples and importantly non-invasive diagnostic specimens like saliva would give better insight into the relevance of miRNA expression ratio as disease predictors. Additional validation in a larger sample cohort would shed more light onto its true strength in oral cancer prognosis.

Defining specific role of miRNAs in cancer is crucial as they control cellular functions by altering target gene expression. Expressional alterations of miRNAs in cancer can hence affect key regulatory pathways. In silico analysis identified SPRR2C, ANXA1, S100A9 as targets of miR-196a-5p and CDC42, RAB22A, EZR as that of miR-204-5p. In Barrett’s esophagus (BE), which is a recognized precursor of esophageal adenocarcinoma (EA), miR-196a was recognized as a potential marker of disease progression with SPRR2C and S100A9 as its targets^39^. MiR-196a is also reported to promote proliferation, invasion and metastasis of EA and HNSCC by targeting ANXA1^39,40^. MiR-204, on the other hand plays tumor suppressive role by targeting CDC42 and RAB22A in nasopharyngeal and renal cell carcinoma respectively^29,41^. In oral cancer, knockdown of lncRNA LEF1-AS1 and upregulation of circRNA_0000140 supressed proliferation and metastasis by inhibiting Hippo signaling pathway^42,43^ which in our in silico analysis was recognized as the major signaling pathway regulated by miR-196a and miR-204. Further functional validation of these miRNA targets is required to identify their potential functional role in OSCC.

Thus, in this study we have looked at the differential expression of miRNAs in OSCC, arriving at a 19 tumor specific signature miRNA expression with remarkable demarcative potential. Further analysis of the patient cohort narrowed down specific expression markers for tumor stage, depth, peri-neural invasion and patient survival, highlighting the specificity and sensitivity of miRNA expression profiles. Up-regulation of miR-196a, miR-21, miR-1237 and downregulation of miR-204, miR-144 classified a poor prognosis group in our patient cohort, strongly associated with patient survival and capacity for disease prediction. Correlation of miRNA expression with external datasets (GEO) mostly corroborated our signature miRNA expression profiles adding confidence, the differences being attributed to ethnic variation. miR-1237 emerged as a novel report in our study, significantly over-expressed in oral tumor samples especially in the early stages (stage I and II) with low tumor depth suggesting a role in tumor initiation and positively associated with tumor recurrence. Combining their individual strengths, miRNA expression ratio boosted predictory power and miR-196a/miR-204 expression ratio emerged the best predictor for disease recurrence and patient survival. Through this study, we confirm the potential of miRNAs expression profiles as strong molecular tools in oral cancer diagnosis and prognosis.

## Methods

### Selection of patients

The cases for the present study were selected from patients attending the out-patient Head and Neck Clinic of Regional Cancer Centre, Thiruvananthapuram. Patients with a histopathologically confirmed diagnosis of oral squamous cell carcinoma with no previous history of any type of treatment for cancer and patients free from chronic systemic diseases were included in the study population. Details such as socio-demographic, occupational, habitual, and clinico-pathological features of lesions in each patient were collected in pro-forma.

### Tissue sample collection

After obtaining the signed informed consent from the eligible patients, incision biopsy of tumor was taken from each patient either during the investigative biopsy procedure or surgical excision of the lesion. Normal mucosa was also collected from patients undergoing oral and maxillofacial surgery for reasons other than cancer. All the collected tissue bits were immediately snap frozen and stored in liquid Nitrogen.

### Patient details

The clinico-pathological features of study subjects are described in Supplementary Table 1. Treatment plan for each patient was arrived at by a joint decision between the radiation oncologist and surgeon at the multidisciplinary clinic or by the institute’s tumor board. In general, early stage cancers are mainly subjected to surgery and followed by radiation if the disease is involved into regional nodes. However, for advanced stage diseases (stages III and IV) radiation and/or chemotherapy together with surgery continue to be the standard regime of care. Clinical follow-up of all these patients after treatment were carried out until death or up to a maximum of 60 months. Criteria for histological diagnosis were based upon WHO guidelines for the histological classification of oral lesions.

### RNA isolation

Total RNA was prepared from tissue samples using miRVana miRNA isolation kit (Ambion, TX, USA) as per manufacturer’s protocol. The quality and quantity of the isolated RNA was checked using Biospec 1601 UV–Vis Spectrophotometer (Shimadzu, Japan).

### miRNA microarray

Agilent Human miRNA Microarray v2.0 (G4470B, Agilent Technologies) was used to identify miRNAs differentially expressed in OSCC. MicroRNA processing was carried out according to the manufacturer’s instructions. Hybridized microarrays were scanned with a DNA microarray scanner (Agilent G2565BA) and features were extracted using the Agilent Feature Extraction (AFE) image analysis tool (version A.9.5.3) with default protocols and settings^44^.

### MiRNA microarray data analysis

Data pre-processing and differential expression analysis were done in R Studio using the Bioconductor AgiMicroRna package^45^. The Total Gene Signal provided by the AFE image analysis software was used for data analysis. Data were normalized between arrays using the quantile method^46^. Hierarchical clustering was carried out using Euclidian distance as the distance metric using hclust algorithm and heatmaps were plotted using heatmap-2 function with the gplots package in R Studio (R Studio team). Gene Ontology (GO) Enrichment Analysis of the miRNA microarray data was performed using DAVID^47^ summarized and visualized using REVIGO^48^. Target prediction was performed with platforms like DIANA-microT-CDS and miRPath v2.0, miRWalk v2.0, MiRTarBase v4 and ComiR for maximum overlap.

### cDNA construction and quantitative real time PCR (qRT-PCR)

cDNA was synthesized by priming with a pool of gene-specific looped primers including the primers of the miRNAs of interest and RNU6B, as a universally-expressed endogenous control (Applied Biosystems, Foster City, CA, USA) as per manufacturer’s specifications. PCR products are amplified from cDNA samples using the TaqMan MicroRNA Assay together with the TaqMan Universal PCR Master Mix (Applied Biosystems, Foster City, CA, USA). Raw CT was normalized to account for inter-sample variation using Global Mean Normalization^13,19^. Relative Quantification of the normalized data was done against control using Livaks and Schmittgen’s 2^−ddCT^ method^49^, to obtain fold change with Microsoft Excel spreadsheet.

### Identification of potential targets for the signature miRNAs

The association between miRNAs and their respective gene targets were computed using level 3 data from The Cancer Genome Atlas (TCGA) datasets in HNSC cohort. TCGA data was accessed and analyzed using UCSC Xena Browser (https://xenabrowser.net). In this cohort, there were 528 patients with primary head and neck cancer. miRNA expression was measured using RNA sequencing (Illumina Hiseq). Mirtarbase was used to obtain the experimentally validated microRNA target genes for all the differentially expressed microRNAs. The genes which showed a negative correlation with microRNA expression in the TCGA HNSC cohort were selected as potential targets. The negatively correlated genes were selected by setting Pearson’s r value ≤ − 0.1 and *p *< 0.05.

### Statistical analysis

A two-tailed Student’s *t* test was used to compare between miRNA expression levels obtained by miRNA microarray and Real-Time PCR followed by Spearman’s Rho and Pearson correlation to select validated miRNAs. The association of these selected miRNAs with various disease parameters was assessed using Cox regression and log-rank tests and a *p* value of < 0.05 was considered statistically significant. Significant pathways association of these signature miRNAs to the patient’s overall and disease-free survival (DFS) was evaluated by Kaplan–Meier Survival Curve analysis with the Log-rank statistic. Receiver operating characteristics (ROC) analysis determined the ability of the signature miRNAs to discriminate between OSCC and normal samples.

### Ethical standards

This study has been cleared by the Institutional Human Ethics Committee of Regional Cancer Centre, Trivandrum, India for collecting the tissue samples from patients (Sanction No. 39/2006). Also the study has been carried out as per the guidelines, “National Ethical Guidelines for Biomedical and Health Research Involving Human Participants-2017” formulated by the ICMR, Government of India.

## Supplementary Information

Supplementary Information

## Data Availability

The high throughput data generated during and/or analyzed during the current study are available in the GEO repository (GEO Submission No. GSE168227) at https://www.ncbi.nlm.nih.gov/geo/query/acc.cgi?acc=GSE168227.
