# Supplementary material for: MiRNA expression profiling and emergence of new prognostic signature for oral squamous cell carcinoma
Source: Sci Rep. 2021 Mar 31;11:7298. doi: 10.1038/s41598-021-86316-w (PMC8012614; doi:10.1038/s41598-021-86316-w)
Supplement: Supplementary file 1 — Supplementary Information [file 41598_2021_86316_MOESM1_ESM.docx]

**MiRNA EXPRESSION PROFILING AND EMERGENCE OF NEW PROGNOSTIC SIGNATURE FOR ORAL SQUAMOUS CELL CARCINOMA**

Christo Rajan^a*^, VG Deepak Roshan^a*^, Imran Khan^b*^, Manasa VG^a^, Iris Himal^a^, Jayasree Kattoor^c^, Shaji Thomas^d^, Paturu Kondaiah^b^ and S Kannan^a^

**SUPPLIMENTARY TABLES:**

**SupplementaryTable 1. Patient demographics**

| **Demographics** | **n** | **%** |
| --- | --- | --- |
| **Age** |  |  |
| **Young** | **35** | **24.3** |
| **Old** | **109** | **75.7** |
| **Gender** |  |  |
| **Male** | **90** | **62.5** |
| **Female** | **54** | **37.5** |
| **Site** |  |  |
| **Tongue** | **83** | **57.6** |
| **Buccal Mucosa** | **61** | **42.4** |
| **Habits** |  |  |
| **Alcohol** | **2** | **1.4** |
| **Chewing** | **29** | **20.1** |
| **Smoking** | **4** | **2.8** |
| **Mixed** | **77** | **53.5** |
| **No habits** | **32** | **22.2** |
| **Tumor grade(cytology)** |  |  |
| **SCC** | **7** | **4.9** |
| **WDSCC** | **31** | **21.5** |
| **MDSCC** | **93** | **64.6** |
| **PDSCC** | **13** | **9** |
| **TNM Stage** |  |  |
| **I+ II (Early)** | **40** | **27.8** |
| **III+IV (Late)** | **104** | **72.2** |
| **Recurrence** |  |  |
| **Positive** | **28** | **19.4** |
| **Negative** | **116** | **80.6** |
| **Peri Neural Invasion(PNI)*** |  |  |
| **Positive** | **13** | **26.5** |
| **Negative** | **36** | **73.4** |
| **Tumor Depth*** |  |  |
| **<1mm** | **12** | **25.3** |
| **1-1.9 mm** | **30** | **63.8** |
| **2-4 mm** | **5** | **10.6** |
| **Survival status** |  |  |
| **Overall >5 years** | **56** | **38.9** |
| **>3 years** | **95** | **66** |
| **Disease free > 5 years** | **40** | **27.8** |
| **> 3 years** | **71** | **49.3** |

***Cases with missing data not shown**

**SCC: Squamous Cell Carcinoma; WDSCC: Well Differentiated SCC; MDSCC: Moderately Differentiated SCC; PDSCC: Poorly Differentiated SCC**

**Supplementary Table 2. Pathways predicted for significant miRNAs using DIANA miR-path for a) up-regulated mRNA list and b) down regulated miRNAs.**

| **KEGG pathway up-miRs** | **p-value** | **Target genes** |
| --- | --- | --- |
| **Glycosphingolipid biosynthesis - lacto and neolacto series** | **<0.001** | **3** |
| **ECM-receptor interaction** | **<0.001** | **6** |
| **Fatty acid metabolism** | **<0.001** | **3** |
| **Endocytosis** | **<0.001** | **17** |
| **TGF-beta signalling pathway** | **<0.001** | **7** |
| **Axon guidance** | **<0.001** | **9** |
| **Steroid biosynthesis** | **0.01** | **1** |
| **ErbB signaling pathway** | **0.02** | **6** |
| **Valine, leucine and isoleucine degradation** | **0.02** | **2** |

**a.**

**b**.

| **KEGG pathway down-miRs** | **p-value** | **target genes** |
| --- | --- | --- |
| **PI3K-Akt signaling pathway** | **<0.001** | **53** |
| **Focal adhesion** | **<0.001** | **36** |
| **Transcriptional misregulation in cancer** | **<0.001** | **34** |
| **Pathways in cancer** | **<0.001** | **52** |
| **Dopaminergic synapse** | **<0.001** | **24** |
| **Lysine degradation** | **<0.001** | **10** |
| **Glutamatergic synapse** | **<0.001** | **22** |
| **Endocrine and other factor-regulated calcium reabsorption** | **<0.001** | **13** |
| **Small cell lung cancer** | **<0.001** | **17** |
| **Ubiquitin mediated proteolysis** | **<0.001** | **24** |
| **MAPK signaling pathway** | **<0.001** | **38** |
| **Gap junction** | **<0.001** | **14** |
| **ErbB signaling pathway** | **<0.001** | **16** |
| **Prostate cancer** | **<0.001** | **15** |
| **Serotonergic synapse** | **<0.001** | **19** |
| **Wnt signaling pathway** | **<0.001** | **25** |
| **GnRH signaling pathway** | **<0.001** | **16** |

**Supplementary Table 3: Details of oral cancer datasets from GEO.**

| **Dataset** | **Tissue** | **n** | **Tumors** | **Normals** | **Country** | **Platform** | **Reference** |
| --- | --- | --- | --- | --- | --- | --- | --- |
| **GSE28100** | **Tongue** | **20** | **17** | **3** | **USA** | **Agilent V3** | **Jung et al.,2012** |
| **GSE31277** | **OSCC** | **32** | **16** | **16** | **Brazil** | **Illumina** | **Severino et al.,2013** |
| **GSE34496** | **OSCC** | **76** | **50** | **26** | **USA** | **Affimetrix** | **unpublished** |

**Supplementary Table 4. Receiver Operating Characteristic (ROC) test statistic for enumeration of disease predictive power**

**Supplementary Table 5: Relationship between the clinic-biological variables and survival endpoints in oral cancer patients**

| Factors |  | Univariate Analysis | | | Multivariate Analysis | | |
| --- | --- | --- | --- | --- | --- | --- | --- |
| Disease Free Survival | |  | | | | | |
|  |  | Good | Poor | Log-Rank p-Value | Coefficient | P-Value | Hazard Ratio |
| Sex | Male  Female | 50  27 | 30  26 | 0.085 | 0.631 | 0.056 | 1.880 |
| T-Stat | 1  2  3  4 | 13  43  12  18 | 7  16  11  20 | 0.370 | 0.373 | 0.248 | 1.452 |
| N-Stat | Not Involved  Involved | 37  49 | 16  35 | 0.500 | -0.096 | 0.802 | 0.908 |
| Composite Stage | 1  2  3  4 | 10  18  37  21 | 3  8  23  20 | 0.354 | 0.059 | 0.902 | 1.061 |
| miR196a/miR204 Ratio | < 0.750  > 0.750 | 24  37 | 25  12 | **0.003** | -0.970 | **0.007** | 0.379 |
| Overall Survival | |  | | | | | |
|  |  | Alive | Dead | Log-Rank p-Value | Coefficient | P-Value | Hazard Ratio |
| Sex | Male  Female | 57  31 | 33  23 | 0.442 | 0.227 | 0.513 | 1.254 |
| T-Stat | 1  2  3  4 | 16  43  12  17 | 5  16  12  21 | **0.003** | 0.446 | 0.158 | 1.562 |
| N-Stat | Not Involved  Involved | 40  48 | 17  37 | 0.124 | 0.413 | 0.434 | 1.511 |
| Composite Stage | 1  2  3  4 | 12  20  36  20 | 2  6  25  21 | **0.045** | 0.009 | 0.985 | 1.009 |
| miR196a/miR204 Ratio | < 0.750  > 0.750 | 30  35 | 21  14 | 0.092 | -0.658 | **0.062** | 0.518 |

**Supplementary Table 6: List of Potential Targets for the Signature miRNAs in Oral Cancer**

| **miRNA** | **Targets** | **Pearson Correlation** | **Sig (2-tailed)** |
| --- | --- | --- | --- |
| miR-101-3p | PTGS2 | -.130 | .003 |
|  | APP | -.314 | <.001 |
|  | RAC1 | -.132 | .003 |
| miR-1237-3p | FAM69C | -.251 | .001 |
|  | PACS2 | -.211 | .005 |
|  | SPOP | -.225 | .003 |
| miR-155-5p | MYO10 | -.350 | <.001 |
|  | KRAS | -.176 | <.001 |
|  | FOXO3 | -.167 | <.001 |
| miR-377-3p | CLN8 | -.165 | .001 |
|  | MAP3K9 | -.248 | <.001 |
|  | TMEM30B | -.246 | <.001 |
| miR-190a-5p | PHLPP1 | -.108 | .019 |
|  | TATDN2 | -.104 | .025 |
|  | SERP1 | -.102 | .026 |
| miR-424-5p | TGFBR3 | -.229 | <.001 |
|  | MYB | -.295 | <.001 |
|  | SMAD3 | -.250 | <.001 |
| miR-411-5p | C15orf40 | -.202 | <.001 |
|  | TARBP2 | -.193 | <.001 |
|  | UQCRB | -.178 | <.001 |
| miR-196a-5p | SPRR2C | -.181 | <.001 |
|  | ANXA1 | -.185 | <.001 |
|  | S100A9 | -.122 | .008 |
| miR-196b-5p | TGFBR2 | -.232 | <.001 |
|  | ETS2 | -.100 | .029 |
|  | IGDCC4 | -.199 | <.001 |
| miR-204-5p | CDC42 | -.325 | <.001 |
|  | RAB22A | -.262 | <.001 |
|  | EZR | -.239 | <.001 |
| miR-21-5p | TGFBR3 | -.289 | <.001 |
|  | TIAM1 | -.269 | <.001 |
|  | ANKRD46 | -.229 | <.001 |
| miR-144-3p | PPIC | -.135 | .003 |
|  | COASY | -.149 | .001 |
|  | DAB2 | -.111 | .015 |
| miR-376a-3p | PARD6B | -.260 | <.001 |
|  | PRKCH | -.151 | .003 |
|  | INA | -.147 | .003 |

**SUPPLIMENTARY FIGURES:**

**Supplementary Figure 1. Supervised hierarchical clustering using signature miRNAs differentiating samples site-wise into distinct Tongue and Buccal mucosa clusters with cut off Fold Change > 1.5 and p.value < 0.01. In the heatmap, samples are labelled with prefix ‘t’ for Tongue and ‘b’ for Buccal Mucosa**

**e**


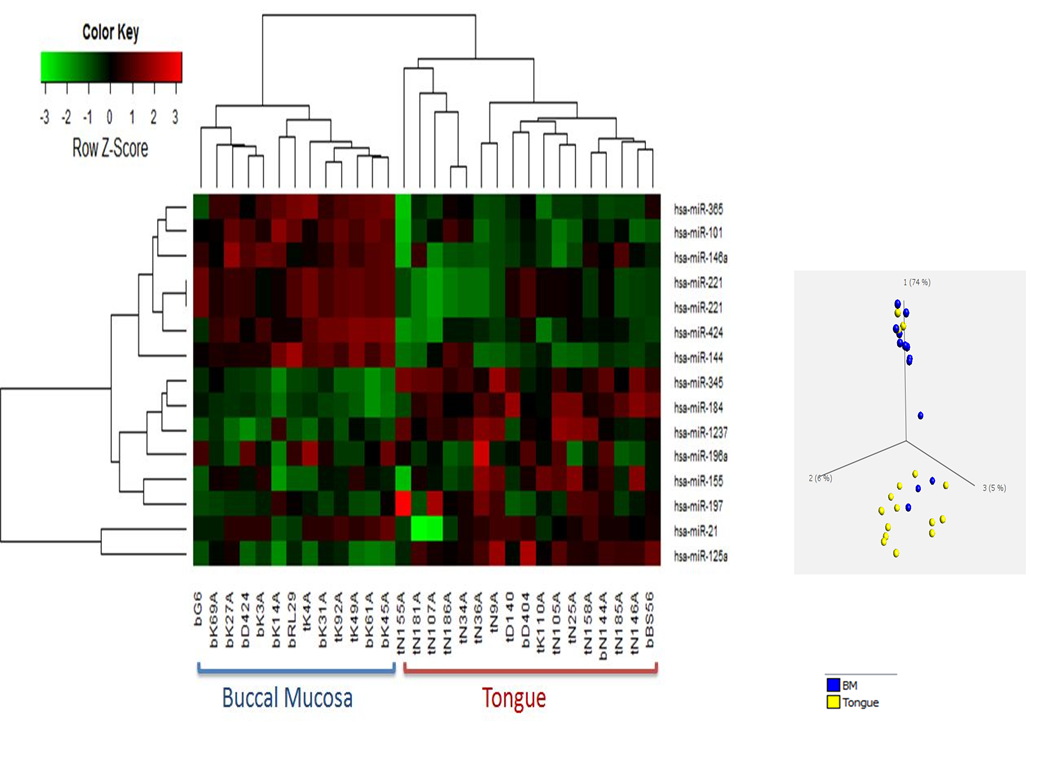


**Supplementary Figure 2. Oral Squamous Cell Carcinoma pathological prognostic factors. a) Peri-Neural Invasion and b) Tumor depth.**

**e**

**
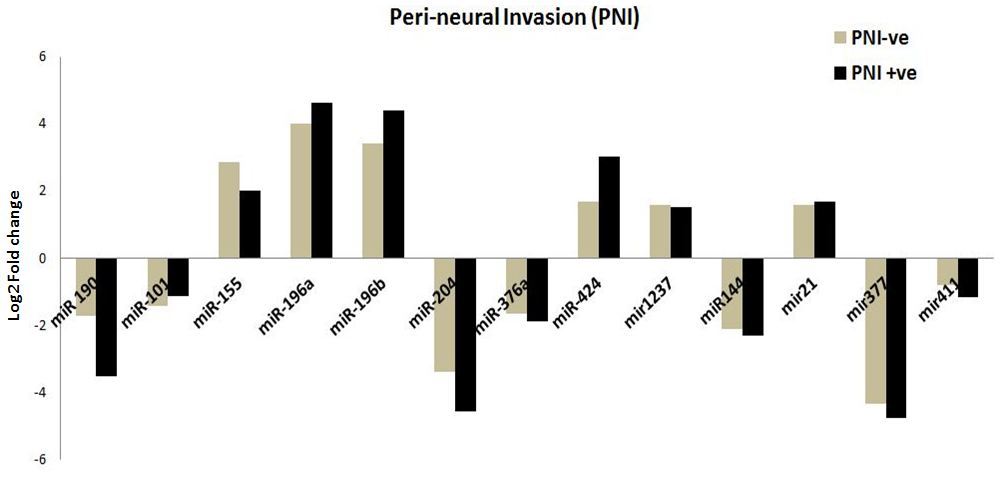
a.**

**b.**

**
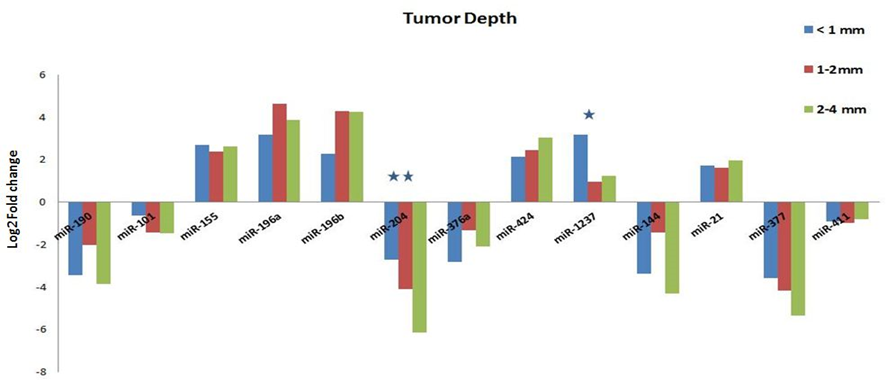
**

**Supplementary Figure 3. Kaplan Meier curve showing Overall and Disease free survival in OSCC patients with respect to expression of a) miR-196a, b)miR-204**

**a.**


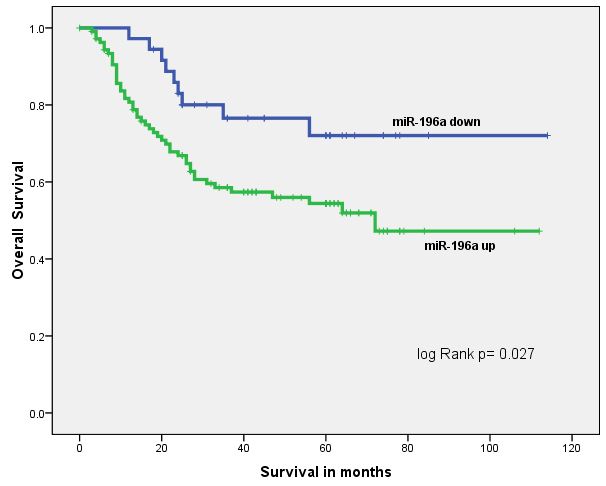

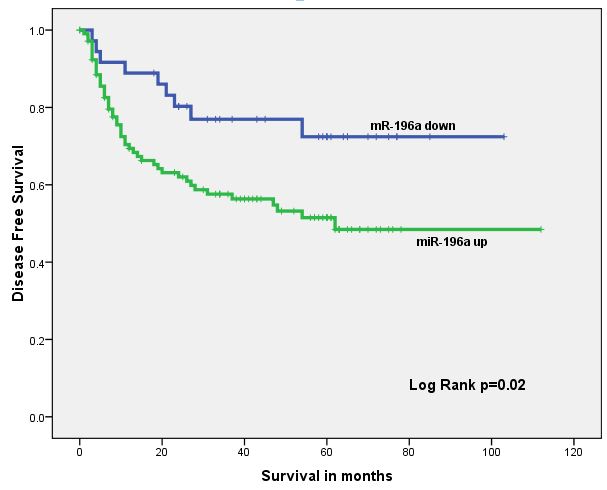


**b.**


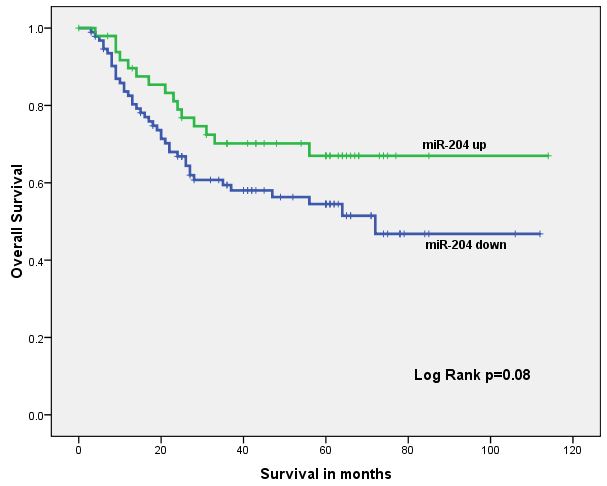

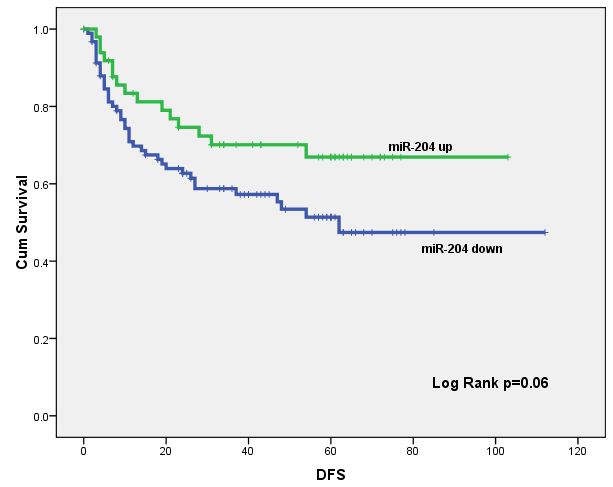


**Supplementary Figure 4. (a) Expression ratio of miR-196a/miR-204 and its association with**

**Overall and Disease-free survival (b) ROC demonstrates the predictory power of miR196a/miR-**

**204expression ratio**

**a.**


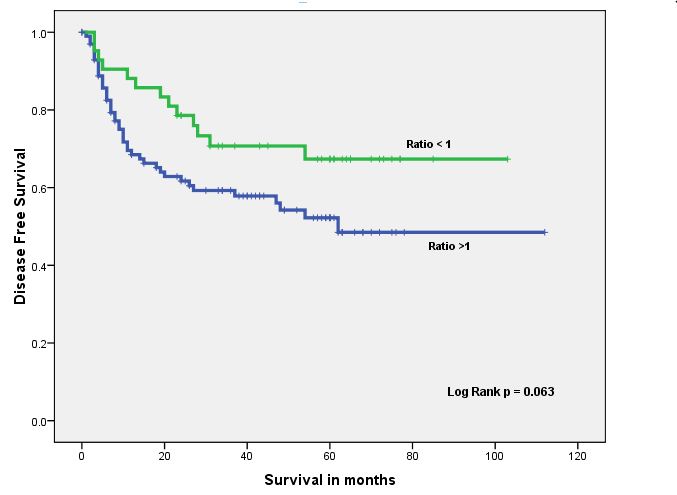

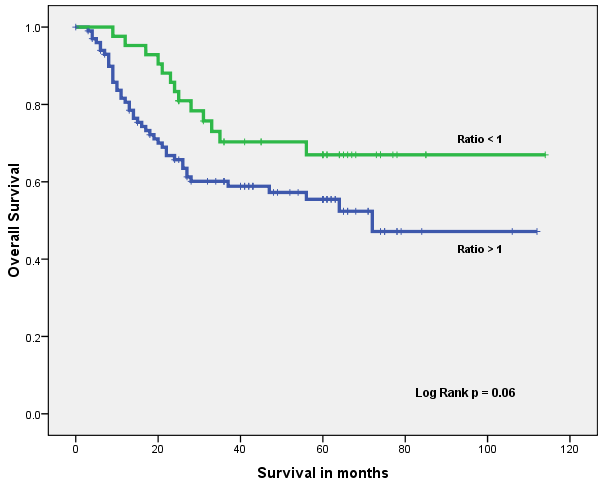


**b.**

**
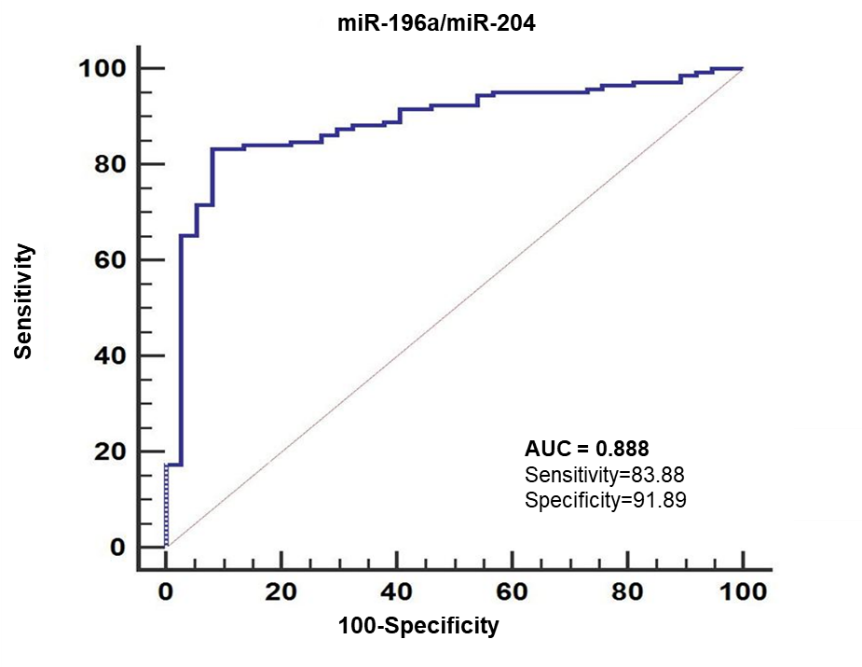
**

**Supplementary Figure 5: MiR-196a/miR-204 target multiple genes in Hippo signaling pathway. Yellow boxes represent target genes of any one of the miRNA, orange boxes represent target genes of more than one miRNAs and green boxes indicates that the genes are not the targets of these miRNA in this context. Figure developed from the output of DIANA miRPath.**

**
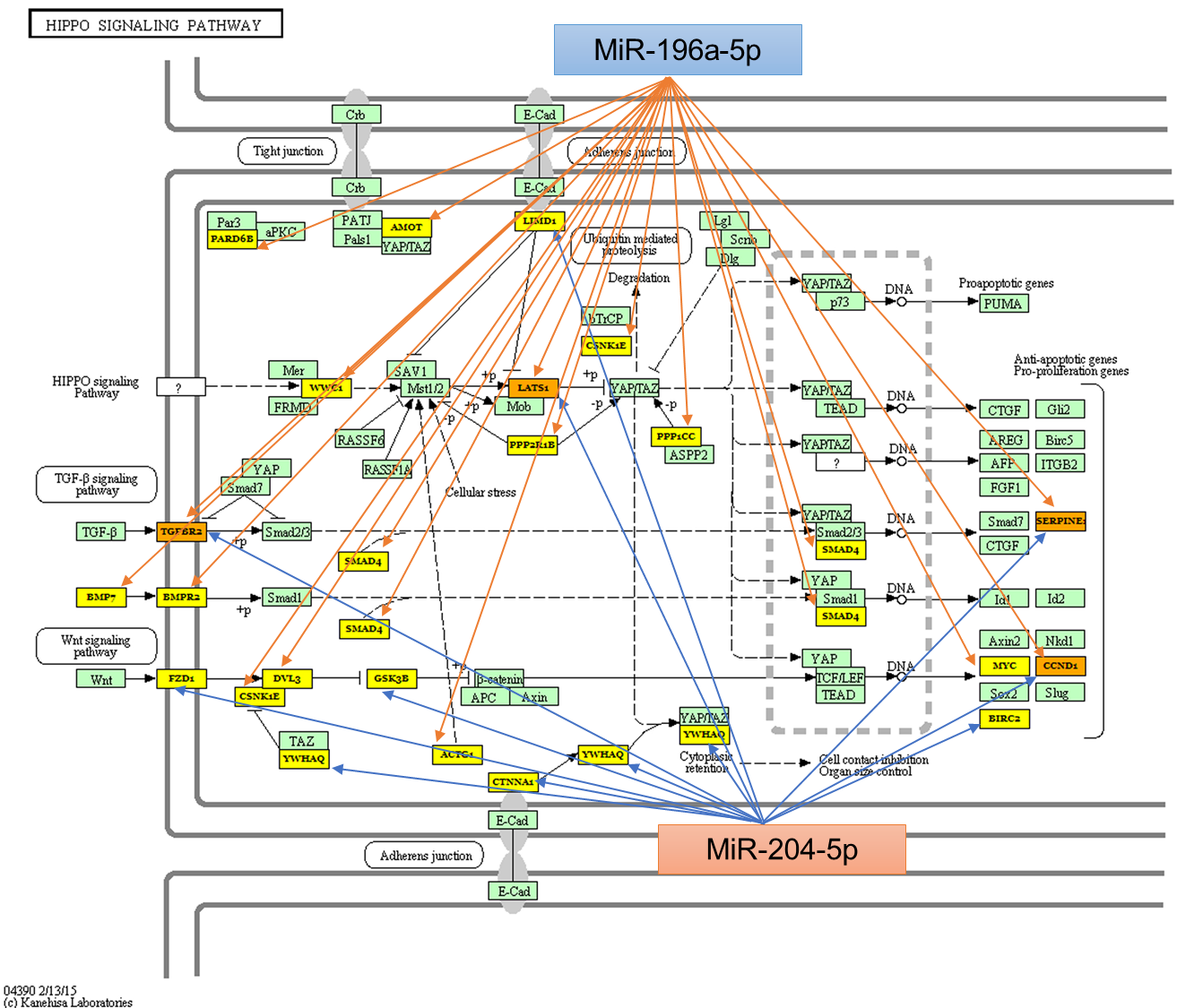
**
